# Efficacy and safety of combining TACE with atezolizumab/bevacizumab versus TACE alone: a retrospective propensity score-matched cohort study in HBV-related hepatocellular carcinoma

**DOI:** 10.3389/fonc.2026.1840779

**Published:** 2026-06-29

**Authors:** Jie Feng, Guocheng Lin, Yeyan Wang

**Affiliations:** 1 Department of Radiology, The Second People's Hospital of China Three Gorges University, Yichang, Hubei, China; 2 Department of Ultrasound Medicine, The Second People's Hospital of China Three Gorges University, Yichang, Hubei, China

**Keywords:** combination therapy, HBV reactivation, HBV-related hepatocellular carcinoma, overall survival, transarterial chemoembolization

## Abstract

**Background:**

Transarterial chemoembolization (TACE) is an established treatment for intermediate to advanced hepatocellular carcinoma (HCC) in China, where chronic hepatitis B virus (HBV) infection constitutes the primary etiology. Although the use of TACE combined with atezolizumab/bevacizumab (TACE+TA) is increasing, robust evidence regarding its efficacy and safety—including the risk of HBV reactivation—remains limited in this etiologically distinct population.

**Methods:**

This retrospective analysis included 246 patients with HBV-related HCC treated between 2020 and 2024. Patients were stratified according to the treatment received: TACE monotherapy (n = 113) or TACE+TA (n = 133). The primary endpoints were overall survival (OS) and progression-free survival (PFS). Tumor response was assessed using the modified Response Evaluation Criteria in Solid Tumors (mRECIST). To minimize selection bias, a 1:1 propensity score matching (PSM) analysis was performed.

**Results:**

After matching, patients receiving TACE+TA achieved superior outcomes compared with TACE monotherapy, with prolonged median OS (19 vs. 13 months; *P* < 0.001) and extended median PFS (8 vs. 6 months; *P* = 0.004). In the unmatched cohort, the objective response rate was also higher in the combination group (48.1% vs. 30.0%; *P* = 0.004). Independent risk factors for worse OS included elevated baseline HBV-DNA load, advanced BCLC stage, and receipt of monotherapy. Notably, the combination therapy group experienced a higher incidence of adverse events, particularly HBV reactivation (8.3% vs. 1.8%; *P* = 0.023), immune-mediated hepatitis, pneumonitis, and thrombocytopenia.

**Conclusions:**

In patients with HBV-related HCC, TACE+TA confers a significant survival advantage over TACE monotherapy, albeit with an increased risk of HBV reactivation and immune-related adverse events. This finding underscores the need for rigorous antiviral prophylaxis and close monitoring in clinical practice.

## Introduction

Primary liver cancer poses a major global health challenge, being one of the most common malignancies worldwide. Notably, China accounts for nearly half (44%) of all liver cancer cases globally. Within the national cancer profile, liver cancer ranks third in incidence and second in mortality, further compounded by a five-year survival rate below 20% (1, 2). Hepatocellular carcinoma (HCC) represents the predominant histological subtype of primary liver cancer worldwide, with chronic hepatitis B virus (HBV) infection serving as a key etiological driver of hepatocarcinogenesis (3). China bears a substantial burden of HBV-related disease; epidemiological studies indicate that 64.09% of patients with primary liver cancer show serological evidence of HBV infection (4). Importantly, the risk of oncogenic progression differs markedly by infection status: approximately 9.6% of acutely infected individuals may develop HCC, compared with 60.2% of chronic HBV carriers (5).

Transarterial chemoembolization (TACE) is the primary treatment method for many patients with intermediate and advanced HCC. This interventional approach involves superselective catheterization of tumor-feeding arteries, followed by embolization of the tumor’s peripheral blood supply and localized delivery of high-concentration chemotherapeutic agents. Through this dual mechanism, TACE aims to induce tumor ischemia while exerting direct cytotoxic effects (6). Nevertheless, the incomplete embolization typical of TACE paradoxically creates a hypoxic tumor microenvironment. This adaptive response is strongly associated with disease recurrence, as reflected in a five-year recurrence rate exceeding 70% (7, 8). Emerging clinical trial data suggest that combining TACE with immune checkpoint inhibitors plus anti-angiogenic agents yields better clinical outcomes than TACE alone in advanced HCC. However, the landmark phase III LEAP-012 trial found that while adding pembrolizumab and lenvatinib to TACE did not significantly improve overall survival (OS), it did lead to a clinically meaningful prolongation of progression-free survival (PFS) in this patient population (9–12).

Although evidence suggests that combining TACE with atezolizumab/bevacizumab (TACE+TA) may improve objective response rates, comparative data on efficacy specifically in HBV-related HCC are currently lacking. To address this gap, the present study directly compares TACE+TA versus TACE monotherapy in patients with HBV-related HCC, aiming to provide an evidence-based foundation for individualized treatment strategies in this population.

## Materials and methods

### Study design and patient selection

Patients diagnosed with HBV-related HCC and treated at our institution between January 2020 and December 2024 were retrospectively reviewed. Based on the treatment received, they were classified into the TACE monotherapy cohort or the TACE+TA cohort.

**Figure 1:**
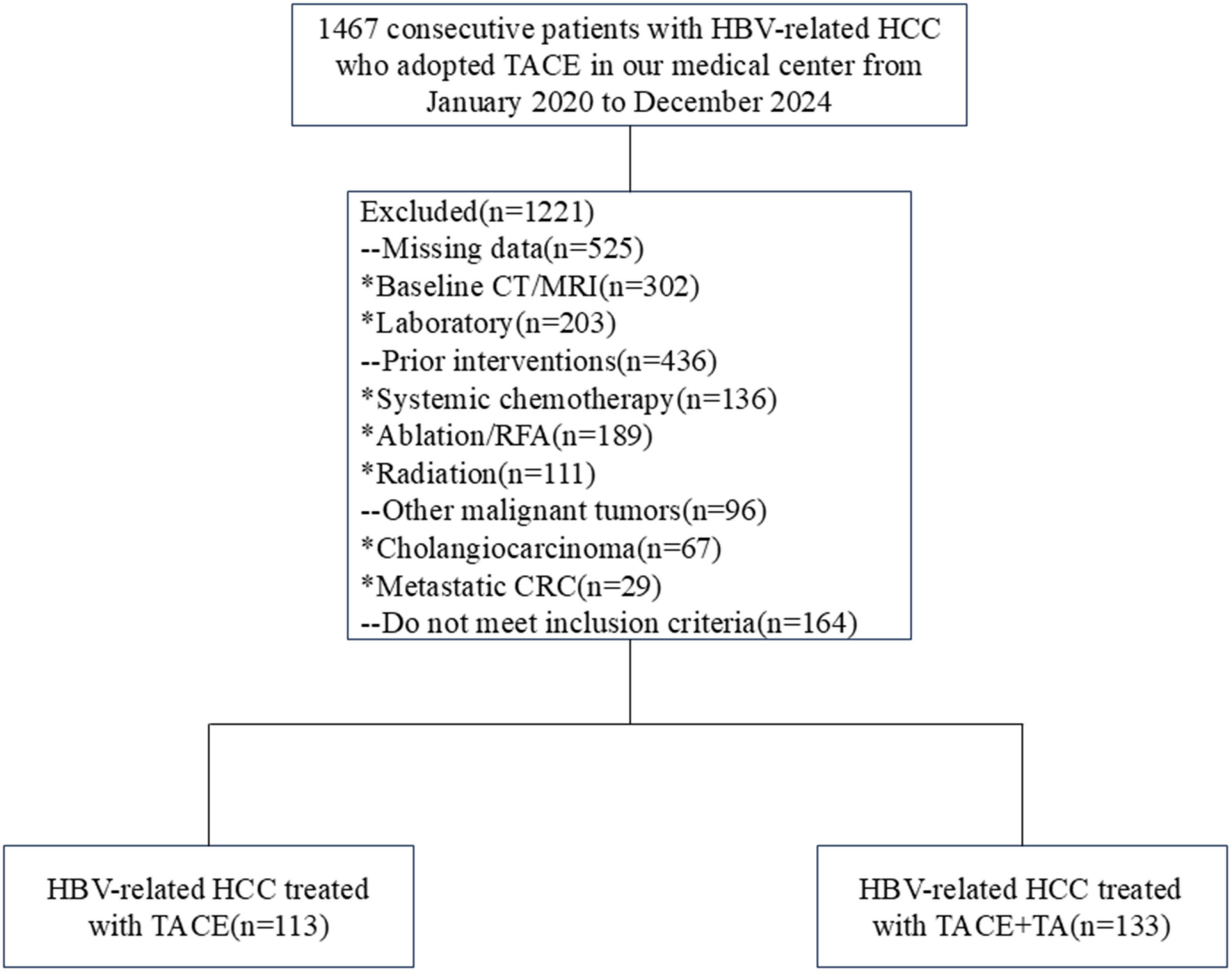
Flow chart showing the screening procedure for HBV-related HCC patients before PSM.

Inclusion criteria were: (1) confirmed HBV-related HCC diagnosis based on histopathology or imaging according to EASL guidelines (13); (2) Barcelona Clinic Liver Cancer (BCLC) stage B or C; (3) Child-Pugh class A or B; (4) Eastern Cooperative Oncology Group (ECOG) performance status 0–1. Exclusion criteria included: (1) incomplete clinical records; (2) ECOG performance status >1; (3) significant comorbidities, including hepatic insufficiency [total bilirubin >3 × upper limit of normal (ULN)], renal impairment [estimated glomerular filtration rate (eGFR) <30 mL/min/1.73 m²], or uncorrectable coagulation disorders [international normalized ratio (INR) >1.5]; (4) concurrent other malignancies, HIV/HCV co-infection, or autoimmune diseases; (5) prior other local or systemic anticancer therapy; (6) loss to follow-up. The patient screening process is summarized in **Figure 1**.

### HBV management

Regardless of baseline HBV-DNA levels, all HBsAg-positive patients were prescribed nucleos(t)ide analogs (NAs) with high genetic barrier to resistance, mainly entecavir (0.5 mg once daily) or tenofovir disoproxil fumarate (300 mg once daily). Antiviral therapy was initiated at least 7 days before the first TACE session and continued uninterrupted throughout the entire treatment period and follow-up period to minimize the risk of HBV reactivation.

### TACE procedure

The TACE procedure was performed by experienced interventional radiologists, each with at least five years of specialized practice. Using a coaxial catheterization technique, a 5-F catheter was advanced to the common hepatic artery, followed by superselective catheterization of the tumor-feeding arteries with a 3-F microcatheter. Subsequently, an emulsion composed of iodized oil (Lipiodol Ultra-Fluid; 2–20 mL) and doxorubicin hydrochloride (20–60 mg) was infused under fluoroscopic guidance. To achieve complete embolization, gelatin sponge particles (300–700 μm) were then administered until angiographic stasis was observed. Post-procedural angiography was performed to confirm tumor devascularization and the absence of non-target embolization.

### Administration of atezolizumab and bevacizumab

In the combination therapy group, patients received intravenous atezolizumab (15 mg/kg) and bevacizumab (15 mg/kg). The first dose was administered 7 ± 3 days after the initial TACE session, avoiding the post-embolization inflammatory phase. Treatment was deferred if TACE-related fever (>38.5 °C) or alanine aminotransferase (ALT) elevation >5 × ULN occurred and was resumed upon resolution of these conditions. Both agents were administered on a fixed 21-day cycle schedule, regardless of subsequent TACE procedures. A predefined protocol guided the management of treatment-related toxicities: for grade ≥3 immune-related adverse events (e.g., hepatitis, pneumonitis), immunotherapy was suspended and glucocorticoids (methylprednisolone 1–2 mg/kg/day) were initiated; in cases of HBV reactivation, antiviral therapy was intensified and immunotherapy permanently discontinued; for significant thrombocytopenia, targeted agents were temporarily withheld and platelet transfusion was provided as clinically indicated.

### Definitions and follow-up

All participants were longitudinally monitored until November 30, 2025. Contrast-enhanced cross-sectional imaging (CT/MRI) and comprehensive laboratory tests, including HBV-DNA quantification, liver function (ALT, AST, total bilirubin, albumin), renal function, and complete blood count, were performed every 6–8 weeks to evaluate treatment response, HBV viral load, and treatment-related toxicities. PFS and OS were compared between the TACE monotherapy and combination therapy cohorts. PFS was defined as the time from treatment initiation to radiologically confirmed tumor progression or last follow-up, while OS represented the duration from therapy commencement to death from any cause or the end of the study. Response evaluation followed the modified Response Evaluation Criteria in Solid Tumors (mRECIST) at 12 ± 2 weeks post-treatment initiation (14). Tumor responses were categorized as complete response (CR), partial response (PR), stable disease (SD), or progressive disease (PD). The objective response rate (ORR) was defined as the proportion of patients achieving CR or PR, and the disease control rate (DCR) was defined as the proportion achieving CR, PR, or SD. Adverse events in both arms were systematically documented using the Common Terminology Criteria for Adverse Events (CTCAE) version 5.0 (15). Serious adverse events were defined as those resulting in death, persistent or significant disability, or requiring hospitalization.

The definition of HBV reactivation is as follows: for patients with detectable baseline HBV-DNA (≥100 IU/mL): an increase in HBV-DNA level by ≥2 log10 IU/mL compared with the baseline level, with or without concomitant elevation of serum aminotransferase levels, and excluding other causes of liver function abnormality (e.g., TACE-related liver injury, immune-mediated hepatitis, drug-induced liver injury). For patients with undetectable baseline HBV-DNA (<100 IU/mL): reappearance of detectable HBV-DNA (≥100 IU/mL) during treatment or follow-up, with or without aminotransferase elevation, after excluding other etiologies of liver injury.

### Statistical analyses

All data were analyzed using SPSS version 29.0 (IBM Corp., Armonk, NY, USA) and R software (version 4.5.2; R Foundation for Statistical Computing). A two-tailed *P* value < 0.05 was considered statistically significant for all tests. Categorical variables were described as frequencies and percentages and compared between groups using Pearson’s chi-square test or Fisher’s exact test (when the expected frequency in any cell was <5). Continuous variables were tested for normality using the Shapiro-Wilk test; normally distributed variables were compared using the independent samples Student’s t-test, and non-normally distributed variables were compared using the Wilcoxon rank-sum test.

Cox proportional hazards model assumptions: The proportional hazards assumption was rigorously validated using the Schoenfeld residual test for all variables included in the final multivariate Cox models. Variables with a *P* value < 0.10 on univariate analysis were entered into a multivariate Cox proportional hazards model using the forward stepwise method.

To reduce potential confounding and selection bias, propensity score matching (PSM) was conducted using 1:1 nearest-neighbor matching without replacement and a caliper width of 0.2. All baseline covariates were entered into the logistic regression model to calculate the propensity score. Specifically, the following variables were included: gender, age, smoking history, drinking history, Child-Pugh grade, BCLC stage, extrahepatic metastasis, vascular invasion, ECOG performance status, tumor size, tumor number, α-fetoprotein level, number of TACE sessions, HBV-DNA load, AST, ALT, total bilirubin, prothrombin time, and albumin. After PSM, 95 pairs of patients (95 in each group) were successfully matched. Standardized mean differences (SMDs) were calculated to evaluate covariate balance before and after matching, with an SMD below 0.1 considered indicative of good balance. After matching, all baseline covariates had SMD < 0.1, indicating good balance between the two groups (**Table 1**). Treatment year and post-progression therapy were included as covariates in the multivariate Cox regression model to adjust for potential era bias and subsequent treatment effects. OS and PFS were estimated using the Kaplan-Meier method, and between-group comparisons were performed using the log-rank test. Hazard ratios (HRs) and 95% confidence intervals (CIs) were derived alongside median survival times.

**Table 1:** Baseline characteristics.

Characteristics	Before PSM					After PSM			
	TACE+TA (n=133)	TACE alone (n=113)		P value	SMD	TACE+TA (n=95)	TACE alone (n=95)	P value	SMD
Gender				0.366	0.116			0.860	0.026
Male	102 (76.7%)	92 (81.4%)				75 (78.9%)	74 (77.9%)		
Female	31 (23.3%)	21 (18.6%)				20 (21.1%)	21 (22.1%)		
Age (years)	56 (19)	62 (17)		0.080	0.194	56 (19)	62 (18)	0.563	0.059
Smoking history				0.706	0.048			0.460	
Yes	82 (61.7%)	67 (59.3%)				59 (62.1%)	54 (56.8%)		
No	51 (38.3%)	46 (40.7%)				36 (37.9%)	41 (43.2%)		
Drinking history				0.105	0.209			0.478	0.063
Yes	58 (43.6%)	61 (54.0%)				44 (46.3%)	47 (49.5%)		
No	75 (56.4%)	52 (46.0%)				55 (57.9%)	48 (50.5%)		
Child-Pugh				0.136	0.192			0.883	0.021
A	82 (61.7%)	59 (52.2%)				56 (58.9%)	55 (57.9%)		
B	51 (38.3%)	54 (47.8%)				39 (41.1%)	40 (42.1%)		
BCLC stage				0.598	0.067			0.653	0.065
B	55 (41.4%)	43 (38.1%)				37 (38.9%)	34 (35.8%)		
C	78 (58.6%)	70 (61.9%)				58 (61.1%)	61 (64.2%)		
Metastasis				0.719	0.046			0.398	0.023
Yes	19 (14.3%)	18 (15.9%)				11 (11.6%)	15 (15.8%)		
No	114 (85.7%)	95 (84.1%)				84 (88.4%)	80 (84.2%)		
Vascular invasion				0.394	0.112			1.000	<0.001
Yes	15 (11.4%)	17 (15.0%)				13 (13.7%)	13 (13.8%)		
No	117 (88.6%)	96 (85.0%)				82 (86.3%)	82 (86.4%)		
ECOG				0.078	0.225			0.574	0.082
0	114 (85.7%)	87 (77.0%)				79 (83.2%)	76 (80.0%)		
1	19 (14.3%)	26 (23.0%)				16 (16.8%)	19 (20.0%)		
Tumor size (cm)	4.0 (4.5)	4.9 (4.2)		0.161	0.141	3.9 (4.1)	4.9 (4.2)	0.147	0.054
Tumor number				0.164	0.179			1.000	<0.001
1	45 (33.8%)	29 (25.7%)				27 (28.4%)	27 (28.5%)		
≥2	88 (66.2%)	84 (74.3%)				68 (71.4%)	68 (71.5%)		
α-fetoprotein level				0.443	0.098			0.755	0.045
<400 ng/mL	51 (38.3%)	38 (33.6%)				29 (30.5%)	31 (32.6%)		
≥400 ng/mL	82 (61.7%)	75 (66.4%)				66 (69.5%)	64 (64.4%)		
TACE session				0.383	0.023			0.788	0.039
1	15 (11.3%)	9 (8.0%)				7 (7.4%)	8 (8.4%)		
≥2	118 (88.7%)	104 (92.0%)				88 (92.6%)	87 (91.6%)		
HBV-DNA				0.095	0.215			1.000	<0.001
<10000 IU/mL	88 (66.2%)	63 (55.8%)				55 (57.9%)	55 (57.9%)		
≥10000 IU/mL	45 (33.8%)	50 (44.2%)				40 (42.1%)	40 (42.1%)		
AST (µmol/L)	48 (38)	46 (38)		0.504	0.148	47 (37.5)	46 (39)	0.963	0.037
ALT (µmol/L)	42 (32)	44 (32)		0.459	0.036	41 (39)	45 (31.5)	0.472	0.022
TB (µmol/L)	19.3 (10.5)	15.3 (10.3)		0.015	0.125	19.3 (11.0)	15.3 (10.3)	0.061	0.008
Alb (g/L)	36.3 (7.4)	36.2 (8.5)		0.636	0.085	35.9 (7.4)	35.6 (8.8)	0.574	0.067
PT (s)	13.9 (1.3)	14.1 (1.9)		0.256	0.038	13.9 (1.4)	14.2 (1.8)	0.247	0.026

PSM, Propensity score matching; TACE, Transarterial chemoembolization; BCLC, Barcelona Clinic Liver Cancer; SMD, Standardized Mean Difference; ECOG, Eastern Cooperative Oncology Group; ALT, Alanine aminotransferase; AST, Aspartate aminotransferase; PT, Prothrombin time; TB, Total bilirubin; Alb: Albumin.

## Results

### Study population and patient characteristics

A total of 246 patients with HBV-related HCC were included in this analysis, of whom 133 were allocated to the TACE+TA group and 113 to the TACE monotherapy group. Baseline demographic and clinical characteristics are shown in **Table 1**. After PSM, 95 pairs of patients were successfully matched. Baseline characteristics were well balanced between the two groups after matching, with all SMDs < 0.1 (**Table 1**). At the final follow-up, 62 deaths (65.3%) had occurred in the TACE+TA group, compared with 72 deaths (75.8%) in the TACE monotherapy group. In the unmatched cohort, 85 deaths (63.9%) occurred in the combination group versus 86 deaths (76.1%) in the monotherapy group.

### Tumor response

Before PSM, the TACE+TA group exhibited significantly better tumor response outcomes compared with the TACE monotherapy group. Specifically, CR was observed in 7 patients (5.2%) versus 2 patients (1.8%), PR in 57 (42.9%) versus 32 (28.3%), and SD in 40 (30.1%) versus 32 (28.3%), respectively. Consequently, the ORR was significantly higher in the combination therapy group (48.1% [64/133]) than in the monotherapy group [30.0% (34/113); *P* = 0.004]. Similarly, the DCR was markedly elevated in the TACE+TA group [78.2% (104/133)] compared with the TACE alone group [58.4% (66/113); *P* = 0.002].

### Prognostic factors affecting OS and PFS

Before PSM, univariate analysis revealed that HBV-DNA load, maximum tumor diameter, tumor multiplicity, extrahepatic metastasis, BCLC stage, and treatment modality were significantly associated with OS, whereas HBV-DNA load, tumor multiplicity, and treatment modality were associated with PFS (**Table 2**). In the multivariate Cox analysis, elevated HBV-DNA load (HR = 2.030, 95% CI: 1.480–2.784, *P* < 0.001), advanced BCLC stage (HR = 2.572, 95% CI: 1.469–4.500, *P* < 0.001), and receipt of monotherapy (HR = 1.769, 95% CI: 1.296–2.416, *P* < 0.001) were independent prognostic factors for worse OS. For PFS, both high HBV-DNA load (HR = 1.384, 95% CI: 1.051–1.823, *P* = 0.021) and monotherapy (HR = 1.493, 95% CI: 1.132–1.968, *P* = 0.005) remained independently associated with shorter survival (**Table 3**).

**Table 2:** Univariate analysis of prognostic factors for OS and PFS before PSM.

Variable	OS			PFS			
	HR	95% CI	P	HR		95% CI	P
Gender Male/Female	0.952	0.656-1.383	0.797	0.920	0.668-1.266		0.607
Age (years)	1.004	0.992-1.016	0.545	1.001	0.991-1.011		0.868
Smoking history Yes/No	0.910	0.670-1.237	0.549	1.018	0.780-1.328		0.896
Drinking history Yes/No	1.004	0.741-1.360	0.979	1.013	0.780-1.316		0.924
ALT	1.001	0.997-1.004	0.698	1.002	0.999-1.005		0.258
AST	1.001	0.998-1.005	0.541	1.001	0.998-1.004		0.482
Alb	0.992	0.965-1.019	0.548	0.987	0.964-1.011		0.290
PT	1.029	0.929-1.140	0.586	1.018	0.932-1.111		0.696
TB	0.999	0.993-1.005	0.709	0.999	0.993-1.004		0.640
Child-Pugh B/A	1.252	0.926-1.693	0.144	1.036	0.796-1.348		0.792
BCLC stage	2.557	1.820-3.591	<0.001	1.308	1.000-1.711		0.050
C/B							
Metastasis Yes/No	1.774	1.120-2.808	0.015	1.211	0.790-1.857		0.379
Vascular invasion Yes/No	0.793	0.508-1.238	0.307	0.897	0.616-1.305		0.569
ECOG 1/0	0.952	0.646-1.403	0.804	0.889	0.636-1.244		0.492
Tumor size	1.047	1.006-1.090	0.025	1.030	0.995-1.067		0.094
Tumor number ≥2/1	2.361	1.612-3.459	<0.001	1.301	0.946-1.736		0.073
α-fetoprotein level	1.164	0.849-1.594	0.346	0.951	0.727-1.246		0.717
≥400/<400							
TACE session	1.310	0.758-2.265	0.333	1.05	0.678-1.642		0.812
≥2/1							
Therapy	2.082	1.525-2.841	<0.001	1.514	1.159-1.978		0.002
Alone/Combined							
HBV-DNA	1.873	1.378-2.545	<0.001	1.416	1.081-1.856		0.012
≥10000/<10000							

OS, Overall survival; PFS, Progression-free survival; HR, Hazard ratio; CI, Confidence interval; ALT, Alanine aminotransferase; AST, Aspartate aminotransferase; PT, Prothrombin time; TB, Total bilirubin; Alb, Albumin; BCLC, Barcelona Clinic Liver Cancer; ECOG, Eastern Cooperative Oncology Group; TACE, Transarterial chemoembolization.

**Table 3:** Multivariate analysis of prognostic factors for OS and PFS before PSM.

Variable	OS				PFS			
	HR		95% CI	P	HR		95% CI	P
BCLC stage		2.572	1.469-4.500	<0.001	1.307	0.826-2.069		0.253
C/B								
Metastasis Yes/No		1.053	0.680-1.629	0.817				
Tumor size		0.990	0.947-1.035	0.666	1.006	0.968-1.045		0.774
Tumor number ≥2/1		1.115	0.605-2.055	0.727	0.971	0.603-1.562		0.903
Therapy		1.769	1.296-2.416	<0.001	1.493	1.132-1.968		0.005
Alone/Combined								
HBV-DNA		2.030	1.480-2.784	<0.001	1.384	1.051-1.823		0.021
≥10000/<10000								

OS, Overall survival; PFS, Progression-free survival; HR, Hazard ratio; CI, Confidence interval; BCLC, Barcelona Clinic Liver Cancer; TACE, Transarterial chemoembolization.

After PSM, Univariate analysis revealed that HBV-DNA load, maximum tumor diameter, tumor multiplicity, extrahepatic metastasis, BCLC stage, and treatment modality were significantly associated with OS, whereas HBV-DNA load, tumor multiplicity, and treatment modality were associated with PFS (**Table 4**). In the multivariate Cox analysis, elevated HBV-DNA load (HR = 2.075, 95% CI: 1.456–2.957, *P* < 0.001), advanced BCLC stage (HR = 2.213, 95% CI: 1.128–4.339, *P* = 0.021), and receipt of monotherapy (HR = 1.883, 95% CI: 1.320–2.686, *P* < 0.001) were independent prognostic factors for worse OS. For PFS, both high HBV-DNA load (HR = 1.536, 95% CI: 1.119-2.109, *P* = 0.008) and monotherapy (HR = 1.573, 95% CI: 1.142–2.167, *P* = 0.006) remained independently associated with shorter survival (**Table 5**).

**Table 4:** Univariate analysis of prognostic factors for OS and PFS after PSM.

Variable	OS			PFS			
	HR	95%CI	P	HR		95%CI	P
Gender Male/Female	0.973	0.636-1.487	0.898	0.972	0.675-1.401		0.881
Age(years)	0.995	0.982-1.008	0.466	0.998	0.987-1.010		0.775
Smoking history Yes/No	0.878	0.620-1.242	0.463	1.062	0.784-1.439		0.698
Drinking history Yes/No	0.900	0.638-1.269	0.546	1.008	0.747-1.360		0.960
ALT	1.002	0.998-1.005	0.378	1.002	0.999-1.005		0.218
AST	1.002	0.998-1.006	0.302	1.004	1.000-1.008		0.068
Alb	0.989	0.959-1.021	0.496	0.990	0.963-1.018		0.477
PT	1.030	0.916-1.159	0.623	1.032	0.936-1.138		0.532
TB	0.997	0.987-1.007	0.595	0.993	0.984-1.002		0.140
Child-Pugh B/A	1.137	0.806-1.603	0.465	0.809	0.597-1.095		0.170
BCLC stage	2.095	1.430-3.069	<0.001	1.215	0.890-1.658		0.221
C/B							
Metastasis Yes/No	1.089	0.669-1.772	0.732	0.871	0.561-1.352		0.538
Vascular invasion Yes/No	0.884	0.546-1.430	0.616	0.866	0.569-1.318		0.502
ECOG 1/0	0.893	0.576-1.384	0.613	0.757	0.515-1.113		0.157
Tumor size	1.039	0.992-1.088	0.109	1.011	0.971-1.054		0.591
Tumor number ≥2/1	1.720	1.133-2.609	0.011	1.091	0.783-1.521		0.608
α-fetoprotein level	1.008	0.701-1.450	0.965	0.867	0.631-1.193		0.382
≥400/<400							
TACE session	1.012	0.514-1.994	0.973	1.193	0.674-2.113		0.544
≥2/1							
Therapy	1.830	1.291-2.595	<0.001	1.559	1.145-2.124		0.005
Alone/Combined							
HBV-DNA	1.696	1.204-2.390	0.003	1.399	1.032-1.896		0.031
≥10000/<10000							

OS, Overall survival; PFS, Progression-free survival; PSM, Propensity score matching; HR, Hazard ratio; CI, Confidence interval; ALT, Alanine aminotransferase; AST, Aspartate aminotransferase; PT, Prothrombin time; TB, Total bilirubin; Alb, Albumin; BCLC, Barcelona Clinic Liver Cancer; ECOG, Eastern Cooperative Oncology Group; TACE, Transarterial chemoembolization.

**Table 5:** Multivariate analysis of prognostic factors for OS and PFS after PSM.

Variable	OS			PFS			
	HR	95%CI	P	HR		95%CI	P
BCLC stage	2.213	1.128-4.339	0.021	1.214	0.704-2.094		0.484
C/B							
Tumor number ≥2/1	1.092	0.520-2.294	0.816	1.022	0.572-1.826		0.942
Therapy	1.883	1.320-2.686	<0.001	1.573	1.142-2.167		0.006
Alone/Combined							
HBV-DNA	2.075	1.456-2.957	<0.001	1.536	1.119-2.109		0.008
≥10000/<10000							

OS, Overall survival; PFS, Progression-free survival; PSM, Propensity score matching; HR, Hazard ratio; CI, Confidence interval; BCLC, Barcelona Clinic Liver Cancer.

### Overall survival and progression-free survival analysis

**Figure 2:**
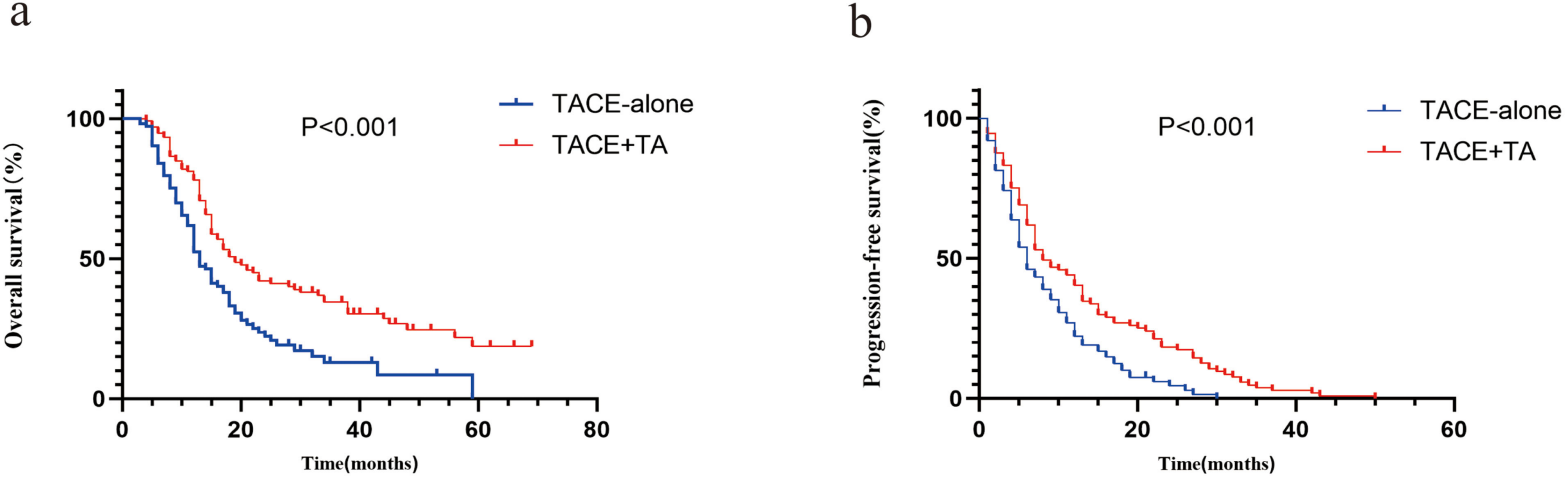
Kaplan-Meier curve of overall survival **(a)** and progression-free survival in **(b)** HBV-related HCC patients before PSM.

Before PSM, the TACE+TA cohort demonstrated significantly prolonged OS compared with the TACE monotherapy group, with a median OS of 19 months (95% CI: 14.5–23.5) versus 13 months (95% CI: 11.2–14.8) (*P* < 0.001; **Figure 2A**). Similarly, PFS was markedly extended in the combination group, with a median PFS of 8 months (95% CI: 5.6–10.4) compared with 6 months (95% CI: 4.6–7.4) in the monotherapy group (*P* < 0.001; **Figure 2B**). After PSM, the TACE+TA cohort similarly showed prolonged OS over the TACE monotherapy group, with a median OS of 19 months (95%CI: 15–33) versus 13 months (95%CI: 12–16) (*P* < 0.001; **Supplementary Figure 1**). PFS was markedly extended in the combination group, with a median PFS of 8 months (95% CI: 7–12) compared with 6 months (95% CI: 5–8) in the monotherapy group (*P* = 0.004; **Supplementary Figure 2**).

**Figure 3:**
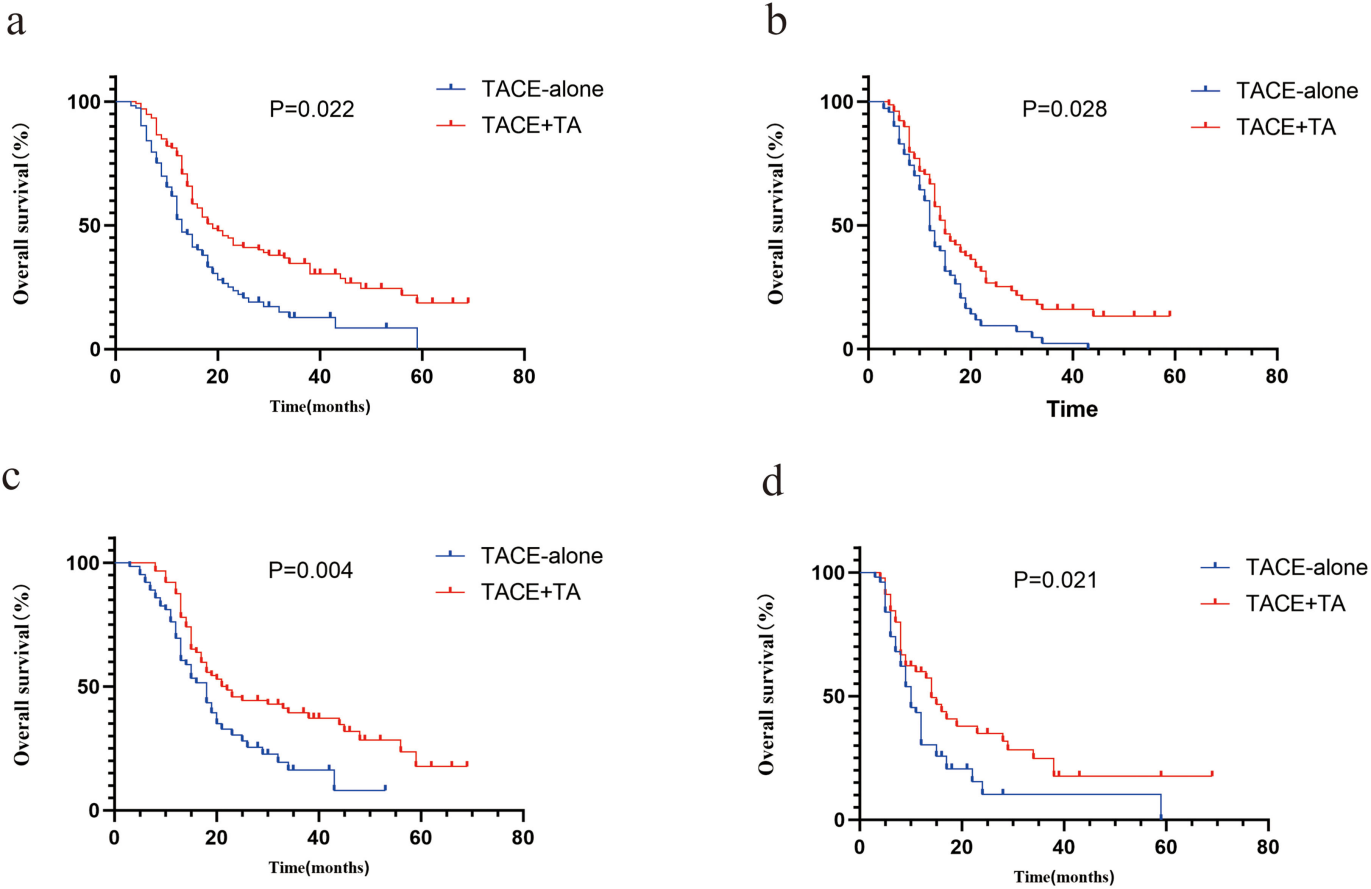
Overall survival comparisons between treatment regimens in subgroup analyses **(a-d)** before PSM.

**Figure 4:**
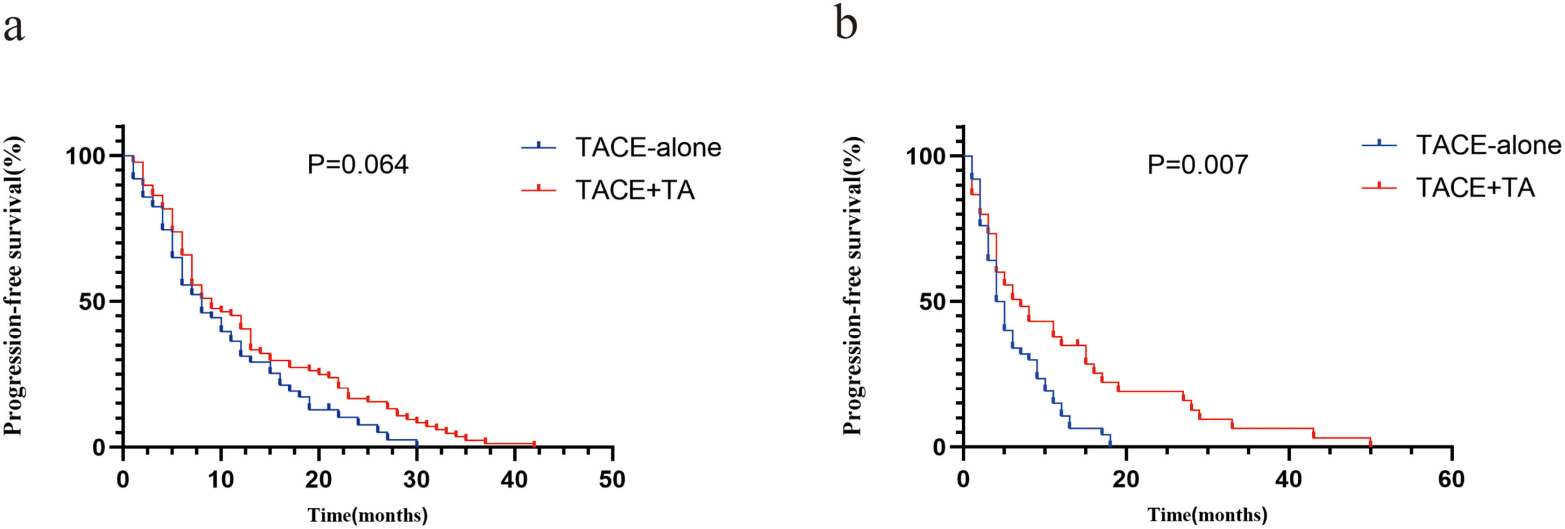
Progression-free survival comparisons between treatment regimens in subgroup analyses **(a, b)** before PSM.

Before PSM, subgroup analyses consistently favored the TACE+TA regimen across all evaluated strata. Among patients with BCLC stage B disease, median OS was 45 months versus 20 months (*P* = 0.022; **Figure 3A**), whereas among those with BCLC stage C disease, median OS was 15 months versus 13 months (*P* = 0.028; **Figure 3B**). Similar survival advantages were observed irrespective of virological status: in the low HBV-DNA load subgroup (<10,000 IU/mL), median OS was 22 months versus 18 months (*P* = 0.004; **Figure 3C**), and in the high HBV-DNA load subgroup (≥10,000 IU/mL), median OS was 14 months versus 10 months (*P* = 0.021; **Figure 3D**). PFS analysis stratified by HBV-DNA load demonstrated that combination therapy significantly improved outcomes only in patients with high viral load. In this subgroup, median PFS was 7 months (95% CI: 3.4–10.4) for TACE+TA versus 4 months (95% CI: 2.8–5.2) for TACE alone (*P* = 0.007; **Figure 4B**). No significant difference was observed in the low HBV-DNA load subgroup (9 vs. 8 months; *P* = 0.064; **Figure 4A**). To further rule out potential era bias and the influence of subsequent treatments on OS, treatment year and post-progression therapy were included as covariates in the multivariate Cox regression model. Treatment year was not an independent prognostic factor for OS (HR = 1.032, 95% CI: 0.918–1.160, *P* = 0.612) or PFS (HR = 1.019, 95% CI: 0.927–1.120, *P* = 0.715). After adjusting for these covariates, TACE monotherapy remained independently associated with worse OS (HR = 1.687, 95% CI: 1.235–2.304, *P* = 0.001), confirming that the observed survival benefit of combination therapy was not confounded by treatment year or post-progression treatment differences (**Supplementary Table 1**).

### Adverse events

All safety analyses were conducted in the unmatched cohort (133 patients in the TACE+TA group and 113 patients in the TACE monotherapy group). Analysis of treatment-associated adverse events revealed a significantly elevated HBV reactivation rate in the combination therapy cohort compared with the TACE monotherapy group (8.3% vs. 1.8%, *P* = 0.023). Following initiation of atezolizumab/bevacizumab, patients in the combination group also demonstrated higher incidences of clinically significant transaminitis requiring intervention (18.0% vs. 8.0%, *P* = 0.021), immune-mediated pneumonitis (5.3% vs. 0%, *P* = 0.013), and treatment-emergent thrombocytopenia (18.0% vs. 8.8%, *P* = 0.037).

Grade 3/4 adverse events: In the TACE+TA group, grade 3/4 adverse events occurred in 14 patients (10.5%). These included thrombocytopenia (5.3%; 3 grade 3, 4 grade 4), hypertension (0.8%; 1 grade 3), immune-mediated pneumonitis (1.5%; 2 grade 3), and elevated transaminase levels (3.0%; 4 grade 3). No grade 3/4 adverse events were reported in the TACE monotherapy group.

Treatment discontinuation: The overall rate of permanent treatment discontinuation due to treatment-related adverse events was 6.0% (8/133) in the TACE+TA group and 1.8% (2/113) in the TACE monotherapy group (*P* = 0.112). The main reasons for discontinuation in the combination group were: immune-mediated pneumonitis (3 cases), HBV reactivation with persistent hepatic dysfunction (2 cases), grade 3 thrombocytopenia (2 cases), and grade 3 hypertension (1 case). All patients who discontinued treatment received appropriate symptomatic management and close follow-up. Complete safety profiles are detailed in **Table 6**.

**Table 6:** Adverse events.

Adverse events	TACE+TA (n=133)	TACE alone (n=113)	P value
HBV reactivation	11 (8.3%)	2 (1.8%)	0.023
Elevated transaminase levels	24 (18.0%)	9 (8.0%)	0.021
Immune-mediated pneumonitis	7 (5.3%)	0 (0%)	0.013
Thrombocytopenia	24 (18.0%)	10 (8.8%)	0.037
Neutropenia	1 (0.8%)	1 (0.9%)	0.941
Anemia	1 (0.8%)	1 (0.9%)	0.941
Bleeding	4 (3.0%)	2 (1.8%)	0.685
Hypertension	8 (6.0%)	1 (0.9%)	0.039
Proteinuria	6 (4.5%)	0 (0%)	0.031

TACE, Transarterial chemoembolization; TACE+TA, TACE plus atezolizumab-bevacizumab.

## Discussion

China carries a significant burden of HBV infection, with a substantial number of patients progressing to HCC through cirrhosis. Notably, approximately 90% of HCC cases in China are etiologically associated with chronic HBV infection (16). Although transarterial TACE combined with immune checkpoint inhibitors plus anti-angiogenic therapy currently represents a cornerstone treatment for advanced HCC, clinical evidence specifically addressing HBV-related HCC remains limited. Importantly, previous studies have identified high baseline HBV-DNA load and immunosuppressive therapy as significant risk factors for HBV reactivation (17), which may lead to hepatic deterioration or compromise treatment efficacy (18). To address this knowledge gap, we conducted a head-to-head comparison evaluating both the efficacy and safety profiles of TACE+TA versus TACE monotherapy in patients with HBV-associated HCC.

Our findings suggest that TACE combined with targeted therapy is associated with superior survival outcomes compared with TACE monotherapy in HBV-associated HCC, as evidenced by a prolonged median overall survival in the unmatched analysis. These results are broadly consistent with previous real-world evidence, such as the cohort reported by You et al., in which combination therapy achieved a median OS of 19 months in patients with portal vein tumor thrombus (19). However, the Chance2211 trial reported higher survival metrics (combination: OS 24.1 months, PFS 15.7 months; monotherapy: OS 15.7 months, PFS 7.7 months) (20). One possible explanation for this discrepancy is that our exclusively HBV-infected cohort may have a distinct viral oncogenic mechanism and a compromised immune microenvironment that could attenuate treatment response—a hypothesis supported by the established role of HBV in modulating PD-L1 expression and promoting T-cell exhaustion (21, 22).

After PSM to minimize selection bias, the survival benefit associated with combination therapy remained evident. In the matched cohort, the TACE+TA group continued to show longer median OS and extended median PFS compared with TACE monotherapy. These post-PSM results strengthen the robustness of the observed associations, although they should still be interpreted as hypothesis-generating rather than definitive evidence given the retrospective design.

Multivariate Cox regression in both the unmatched and matched cohorts consistently identified high pretreatment HBV-DNA load, TACE monotherapy, and advanced BCLC stage as independent predictors of shortened OS. In addition, elevated HBV-DNA load and TACE monotherapy were significantly associated with an increased risk of disease progression. These findings align with the established literature on prognostic factors in HBV-associated HCC (23, 24). It is worth noting that BCLC stage was not significantly associated with PFS in our analysis. A possible explanation—though speculative—is that disease progression in this setting may be driven largely by intrahepatic events regardless of initial BCLC stage, whereas the higher tumor invasiveness and extrahepatic burden in stage B/C disease may ultimately constrain PFS through systemic dissemination.

Despite the favorable survival associations observed with combination therapy, the safety analysis revealed a higher rate of HBV reactivation in the TACE+TA cohort compared with TACE monotherapy. This finding is broadly consistent with previous reports: Zhang et al. documented a 5.3% incidence of HBV reactivation in HBsAg-positive HCC patients receiving PD-1/PD-L1 inhibitors, while He et al. identified high baseline HBV-DNA load as a key risk factor (25, 26). The somewhat higher reactivation rate in our combination group (8.3%) may reflect the additional impact of TACE, which can alter the immune microenvironment and involve chemotherapeutic agents that might themselves contribute to HBV reactivation (27). Nevertheless, because of the retrospective and exploratory nature of this comparison, these safety signals should be considered hypothesis-generating and require confirmation in prospective studies.

Several methodological limitations merit consideration. First, the retrospective, single-center design, coupled with a moderate sample size, may constrain the statistical power and generalizability of our results. Patient grouping was based on historical medical records, which could introduce bias due to varying treatment preferences among physicians at the time of treatment selection. Second, although PSM helped to reduce measured confounding, the possibility of residual or unmeasured confounding cannot be excluded. Third, the combination therapy cohort exclusively received atezolizumab plus bevacizumab, which limits the generalizability of the findings to other immune checkpoint inhibitors or targeted agents. Furthermore, the inclusion of both conventional TACE and drug-eluting bead TACE procedures may have introduced technical heterogeneity into the treatment effects. We acknowledge that the subgroup findings are exploratory in nature, and the differential treatment effects observed in certain subgroups (e.g., by HBV-DNA load) need to be further validated in large-scale, prospective randomized controlled trials.

## Conclusion

In conclusion, our findings suggest that TACE+TA may provide survival benefits for patients with HBV-related HCC, particularly among those with BCLC stage C disease and high HBV-DNA loads. However, given the retrospective design, these results should be viewed as hypothesis-generating rather than practice-changing. The combination regimen was associated with increased risks of HBV reactivation and transaminitis, though these adverse events appeared manageable with appropriate pharmacological interventions. Prospective randomized trials are warranted to definitively establish the risk–benefit profile of this integrated therapeutic strategy in this clinically challenging patient population.

## Data Availability

The raw data supporting the conclusions of this article will be made available by the authors, without undue reservation.

## References

[1] Sung H Ferlay J Siegel RL Laversanne M Soerjomataram I Jemal A . Global Cancer Statistics 2020: GLOBOCAN Estimates of Incidence and Mortality Worldwide for 36 Cancers in 185 Countries. CA: A Cancer J For Clin. (2021) 71:209–49. doi: 10.3322/caac.21660 33538338

[2] Bray F Ferlay J Soerjomataram I Siegel RL Torre LA Jemal A . Global cancer statistics 2018: GLOBOCAN estimates of incidence and mortality worldwide for 36 cancers in 185 countries. CA: A Cancer J For Clin. (2018) 68:394–424. doi: 10.3322/caac.21492 30207593

[3] Vogel A Meyer T Sapisochin G Salem R Saborowski A . Hepatocellular carcinoma. Lancet. (2022) 400:1345–62. doi: 10.1016/S0140-6736(22)01200-4 36084663

[4] Wen B Te L Bai C Jiang W Zuo D Hao Q . Relative contribution of hepatitis B and C viruses in primary liver cancer in China: A systematic review and meta-analysis. J Infect. (2024) 89:106298. doi: 10.1016/j.jinf.2024.106298 39368639

[5] Hwang JP Feld JJ Hammond SP Devena E Alston-Johnson DE Cryeret DR . Hepatitis B virus screening and management for patients with cancer prior to therapy: ASCO provisional clinical opinion update. J Clin Oncol. (2020) 38:3698–715. doi: 10.1200/JCO.20.01757 32716741 PMC11828660

[6] Forner A Gilabert M Bruix J Raoul J-L . Hepatocellular carcinoma. Nat Rev Clin Oncol. (2014) 11:525–35. doi: 10.1016/s1470-2045(15)00014-5 25091611

[7] Maluccio M Covey A . Recent progress in understanding, diagnosing, and treating hepatocellular carcinoma. CA Cancer J Clin. (2012) 62:394–9. doi: 10.3322/caac.21161 23070690

[8] Tan J Fan W Liu T Zhu B Liu Y Wang S . TREM2+ macrophages suppress CD8+ T-cell infiltration after transarterial chemoembolisation in hepatocellular carcinoma. J Hepatol. (2023) 79:126–40. doi: 10.1016/j.jhep.2023.02.032 36889359

[9] Kudo M Ren Z Guo Y Han G Lin H Zheng J . Transarterial chemoembolisation combined with lenvatinib plus pembrolizumab versus dual placebo for unresectable, non-metastatic hepatocellular carcinoma (LEAP-012): a multicentre, randomised, double-blind, phase 3 study. Lancet. (2025) 405:203–15. doi: 10.1016/S0140-6736(24)02575-3 39798578

[10] Sangro B Kudo M Erinjeri JP Qin S Ren Z Chan S . Durvalumab with or without bevacizumab with transarterial chemoembolisation in hepatocellular carcinoma (EMERALD-1): a multiregional, randomised, double-blind, placebo-controlled, phase 3 study. Lancet. (2025) 405:216–32. doi: 10.1016/s0140-6736(24)02551-0 39798579 PMC12282661

[11] Zhang TQ Geng ZJ Zuo MX Li JB Huang JH Huang ZL . Camrelizumab (a PD-1 inhibitor) plus apatinib (an VEGFR-2 inhibitor) and hepatic artery infusion chemotherapy for hepatocellular carcinoma in Barcelona Clinic Liver Cancer stage C (TRIPLET): a phase II study. Signal Transduct Tar. (2023) 8:413. doi: 10.1038/s41392-023-01663-6 37884523 PMC10603153

[12] Galle PR Finn RS Qin SK Ikeda M Zhu AX Kim TY . Patient-reported outcomes with atezolizumab plus bevacizumab versus sorafenib in patients with unresectable hepatocellular carcinoma (IMbrave150): an open-label, randomised, phase 3 trial. Lancet Oncol. (2021) 22:991–1001. doi: 10.1016/s1470-2045(21)00151-0 34051880

[13] EASL Clinical Practice Guidelines . Management of hepatocellular carcinoma. J Hepatol. (2018) 69:182–236. doi: 10.1016/j.jhep.2018.03.019 29628281

[14] Lencioni R Llovet JM . Modified RECIST (mRECIST) assessment for hepatocellular carcinoma. Semin Liver Dis. (2010) 30:52–60. doi: 10.1055/s-0030-1247132 20175033 PMC12268942

[15] Gaba RC Lokken RP Hickey RM Lipnik AJ Lewandowski RJ Salem R . Quality improvement guidelines for transarterial chemoembolization and embolization of hepatic Malignancy. JVIR. (2017) 28:1210–23. doi: 10.1016/j.jvir.2017.04.025 28669744

[16] Seto WK Lo YR Pawlotsky JM Yuen MF . Chronic hepatitis B virus infection. Lancet. (2018) 392:2313–24. doi: 10.1016/S0140-6736(18)31865-8 30496122

[17] Shouval D Shibolet O . Immunosuppression and HBV reactivation. Semin Liver Dis. (2013) 33:167–77. doi: 10.1055/s-0033-1345722 23749673

[18] Huang G Lai EC Lau WY Zhou WP Shen F Pan Z . Posthepatectomy HBV reactivation in hepatitis B-related hepatocellular carcinoma influences postoperative survival in patients with preoperative low HBV-DNA levels. Ann Surg. (2013) 257:490–505. doi: 10.1097/SLA.0b013e318262b218 22868358

[19] You R Cheng Y Diao L Wang C Leng B Yu Z . Immune-targeted therapy with or without transarterial chemoembolization (TACE) for advanced hepatocellular carcinoma with portal vein tumor thrombosis (PVTT): a multicenter retrospective study. Biomedicines. (2024) 12:2124. doi: 10.3390/biomedicines12092124 39335637 PMC11429150

[20] Jin ZC Zhong BY Chen JJ Zhu HD Sun JH Yin GW . Real-world efficacy and safety of TACE plus camrelizumab and apatinib in patients with HCC (CHANCE2211): a propensity score matching study. Eur Radiol. (2023) 33:8669–81. doi: 10.1007/s00330-023-09754-2 37368105 PMC10667391

[21] Rossi M Vecchi A Tiezzi C Barili V Fisicaro P Penna A . Phenotypic CD8 T cell profiling in chronic hepatitis B to predict HBV-specific CD8 T cell susceptibility to functional restoration *in vitro*. Gut. (2023) 72:2123–37. doi: 10.1136/gutjnl-2022-327202 36717219 PMC10579518

[22] Li H Zhai N Wang Z Song H Yang Y Cui A . Regulatory NK cells mediated between immunosuppressive monocytes and dysfunctional T cells in chronic HBV infection. Gut. (2017) 67:2035–44. doi: 10.1136/gutjnl-2017-314098 28899983 PMC6176520

[23] Yan L Ren Y Qian K Kan X Zhang H Chen L . Superselective transarterial chemoembolization for unresectable or "ablation unsuitable" hepatocellular carcinoma in the caudate lobe: a real world, single-center retrospective study. Front Oncol. (2021) 11:678847. doi: 10.3389/fonc.2021.678847 34778023 PMC8581471

[24] Jiang N Zhong B Huang J Li W Zhang S Zhu X . Transarterial chemoembolization combined with molecularly targeted agents plus immune checkpoint inhibitors for unresectable hepatocellular carcinoma: a retrospective cohort study. Front Immunol. (2023) 14:1205636. doi: 10.3389/fimmu.2023.1205636 37583693 PMC10425157

[25] Zhang X Zhou Y Chen C Fang W Cai X Zhang X . Hepatitis B virus reactivation in cancer patients with positive hepatitis B surface antigen undergoing PD-1 inhibition. J Immunother Cancer. (2019) 7:322. doi: 10.1186/s40425-019-0808-5 31753012 PMC6873745

[26] He MK Peng C Zhao Y Liang R Lai Z Kan A . Comparison of HBV reactivation between patients with high HBV-DNA and low HBV-DNA loads undergoing PD-1 inhibitor and concurrent antiviral prophylaxis. Cancer Immunol Immun. (2021) 70:3207–16. doi: 10.1007/s00262-021-02911-w 33813646 PMC10992748

[27] Liu J Sun B Zou J Zhang X Li H Hu H . HBV reactivation and its management in HBV-related HCC patients undergoing HAIC-TKI-ICI therapy: a multicenter study. iScience. (2025) 28:112689. doi: 10.1016/j.isci.2025.112689 40510114 PMC12159918

