# Supplementary material for: Efficacy and safety of combining TACE with atezolizumab/bevacizumab versus TACE alone: a retrospective propensity score-matched cohort study in HBV-related hepatocellular carcinoma
Source: Front Oncol. 2026 Jun 29;16:1840779. doi: 10.3389/fonc.2026.1840779 (PMC13357207; doi:10.3389/fonc.2026.1840779)
Supplement: Supplementary file 1 [file DataSheet1.docx]

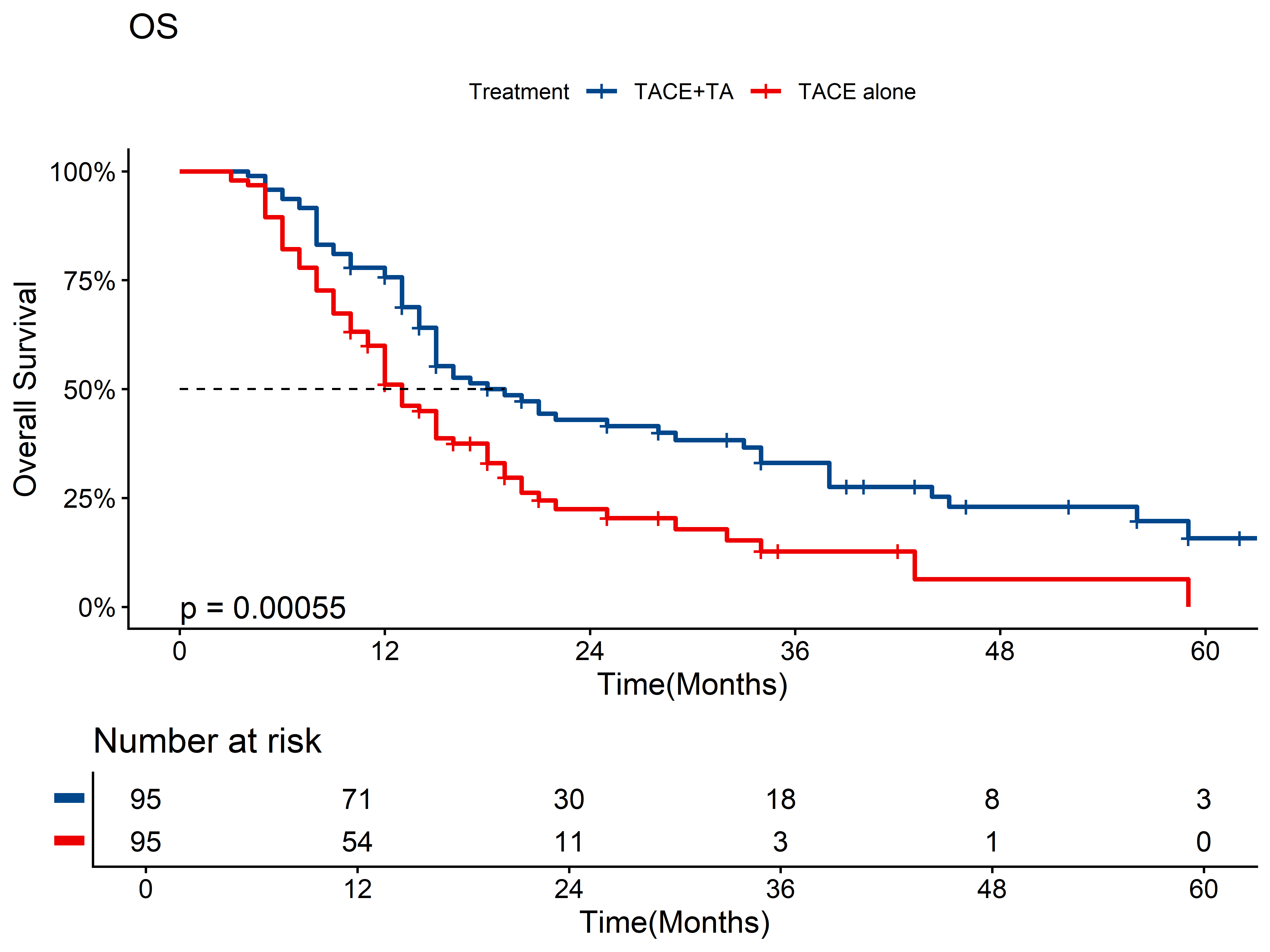


Supplementary Figure 1 Kaplan–Meier curve of overall survival in HBV-related HCC patients after PSM


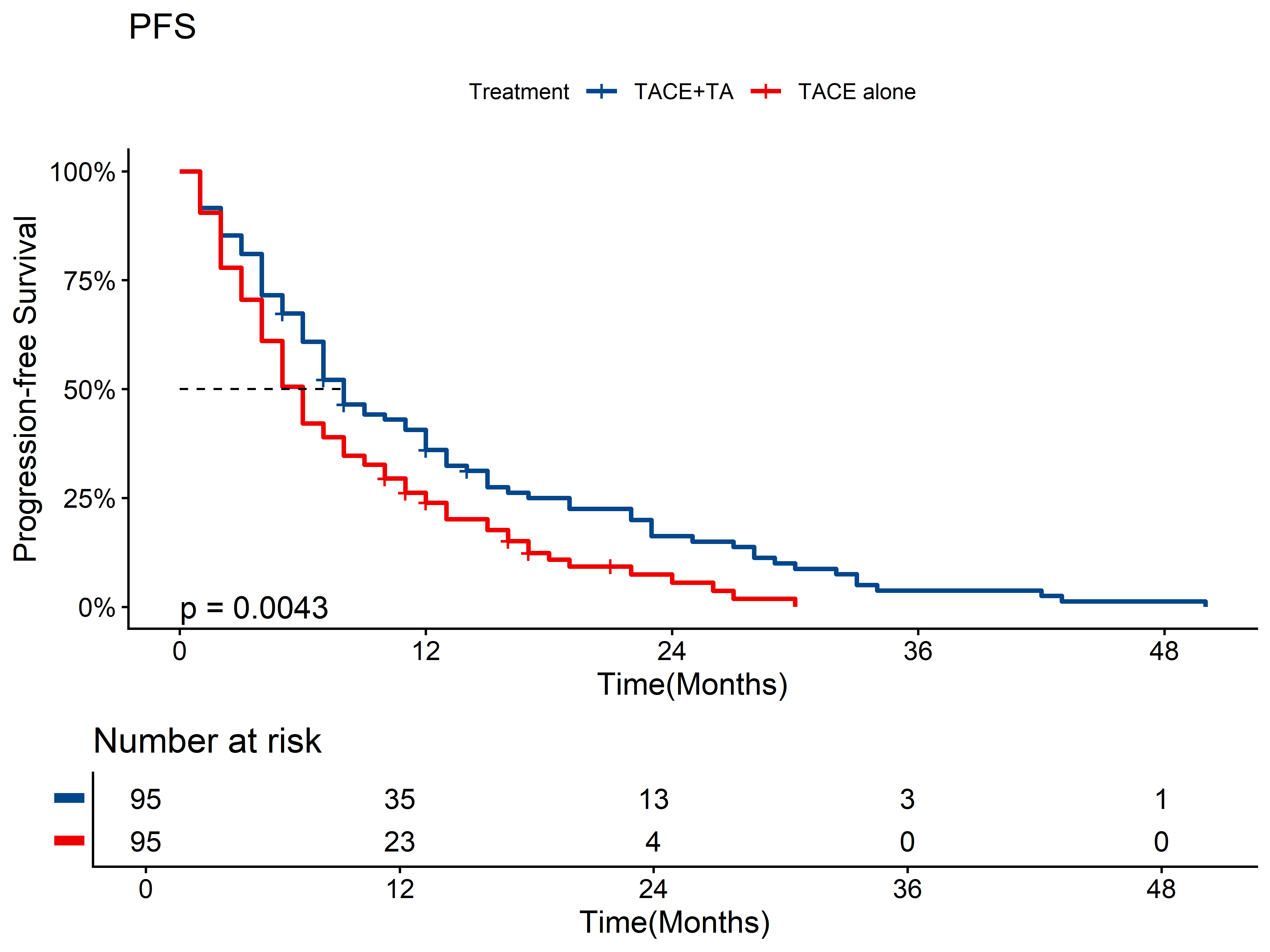


Supplementary Figure 2 Kaplan–Meier curve of progression-free survival in HBV-related HCC patients after PSM

Supplementary Table 1

| Variable | OS |  | PFS |  |
| --- | --- | --- | --- | --- |
|  | HR (95% CI) | P value | HR (95% CI) | P value |
| TACE monotherapy (vs. TACE+TA) | 1.687 (1.235–2.304) | 0.001 | 1.493 (1.132–1.968) | 0.005 |
| Treatment year (per 1-year increase) | 1.032 (0.918–1.160) | 0.612 | 1.019 (0.927–1.120) | 0.715 |
| Post-progression systemic therapy (yes vs. no) | 1.128 (0.856–1.487) | 0.394 | 1.092 (0.874–1.365) | 0.441 |
| High HBV-DNA load (≥10,000 vs. <10,000 IU/mL) | 2.030 (1.480–2.784) | <0.001 | 1.384 (1.051–1.823) | 0.021 |
| Advanced BCLC stage (C vs. B) | 2.572 (1.469–4.500) | <0.001 | 1.307 (0.826–2.069) | 0.253 |

OS: Overall survival; PFS: Progression‑free survival; HR: Hazard ratio; CI: Confidence interval; TACE: Transarterial chemoembolization; BCLC: Barcelona Clinic Liver Cancer.
